# Observing copepods through a genomic lens

**DOI:** 10.1186/1742-9994-8-22

**Published:** 2011-09-20

**Authors:** James E Bron, Dagmar Frisch, Erica Goetze, Stewart C Johnson, Carol Eunmi Lee, Grace A Wyngaard

**Affiliations:** 1 Institute of Aquaculture, University of Stirling, Stirling FK9 4LA, Scotland, UK; 2 University of Oklahoma Biological Station, Kingston, OK 73439, USA; 3 Department of Oceanography, School of Ocean and Earth Science and Technology, University of Hawaii at Manoa, Honolulu, Hawaii 96822, USA; 4 Fisheries and Oceans Canada, Pacific Biological Station, Nanaimo, BC, V9T 6N7, Canada; 5 Center of Rapid Evolution (CORE), University of Wisconsin, Madison, WI, USA; 6 Department of Biology, James Madison University, Harrisonburg, VA, 22807, USA

**Keywords:** genome organization, ecogenomics, parasitism and symbiosis, biological invasion, diapause, response to environmental change

## Abstract

**Background:**

Copepods outnumber every other multicellular animal group. They are critical components of the world's freshwater and marine ecosystems, sensitive indicators of local and global climate change, key ecosystem service providers, parasites and predators of economically important aquatic animals and potential vectors of waterborne disease. Copepods sustain the world fisheries that nourish and support human populations. Although genomic tools have transformed many areas of biological and biomedical research, their power to elucidate aspects of the biology, behavior and ecology of copepods has only recently begun to be exploited.

**Discussion:**

The extraordinary biological and ecological diversity of the subclass Copepoda provides both unique advantages for addressing key problems in aquatic systems and formidable challenges for developing a focused genomics strategy. This article provides an overview of genomic studies of copepods and discusses strategies for using genomics tools to address key questions at levels extending from individuals to ecosystems. Genomics can, for instance, help to decipher patterns of genome evolution such as those that occur during transitions from free living to symbiotic and parasitic lifestyles and can assist in the identification of genetic mechanisms and accompanying physiological changes associated with adaptation to new or physiologically challenging environments. The adaptive significance of the diversity in genome size and unique mechanisms of genome reorganization during development could similarly be explored. Genome-wide and EST studies of parasitic copepods of salmon and large EST studies of selected free-living copepods have demonstrated the potential utility of modern genomics approaches for the study of copepods and have generated resources such as EST libraries, shotgun genome sequences, BAC libraries, genome maps and inbred lines that will be invaluable in assisting further efforts to provide genomics tools for copepods.

**Summary:**

Genomics research on copepods is needed to extend our exploration and characterization of their fundamental biological traits, so that we can better understand how copepods function and interact in diverse environments. Availability of large scale genomics resources will also open doors to a wide range of systems biology type studies that view the organism as the fundamental system in which to address key questions in ecology and evolution.

## Background

The copepods are an extremely ancient group, likely having diverged from other arthropod taxa between 388-522 million years ago [1]. They are also an extraordinarily diverse group with respect to their morphologies, physiologies, life-strategies and habitat preferences, with adult sizes ranging from < 0.1 mm-23 cm. Genomics, defined as the study of genome structure and composition as well as the study of gene expression and function (transcriptomics), has been underutilized in studies of copepods. Although over 12000 validated species of copepods have been recognised to date, there are only modest sequence resources for copepods in public databases. To date, sequencing efforts and the application of genomic techniques have been limited to a small number of species in the orders: Harpacticoida, Calanoida, Cyclopoida, and Siphonostomatoida with estimated species numbers of 7288, 4937, 3241 and 3348, respectively [2]. In this article we discuss why new investments in copepod genomic research are warranted and illustrate how the development of genomics resources for copepods will enable researchers to address key questions related to environmental and ecosystem health, the sustainability of fisheries, evolution, symbiosis and parasitism, biological invasion, and speciation.

### The global importance of copepods

**Figure 1:**
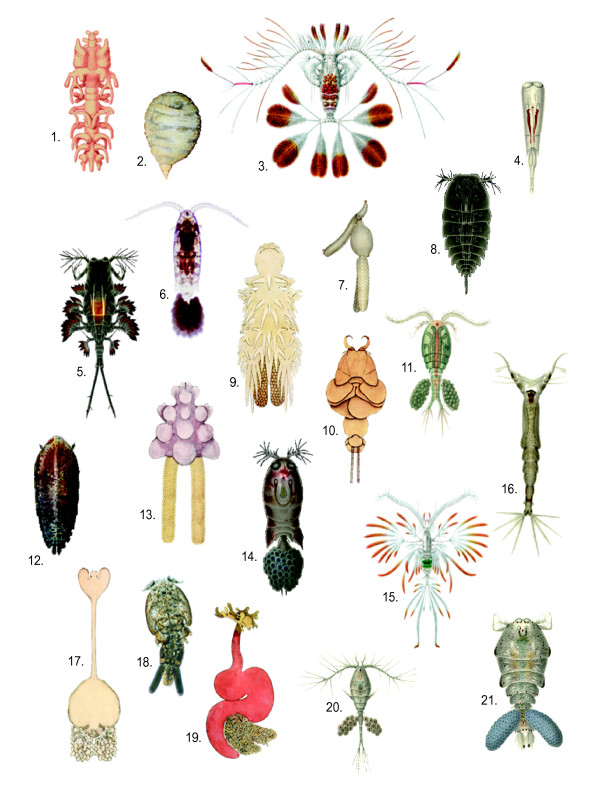
**Illustration showing diversity of copepod forms**. 1. *Philichthys xiphiae *2. *Sarcotaces sp*. 3. *Calocalanus pavo *4. *Farranula rostrata *5. *Copilia vitrea *6. *Paracalanus parvus *7. *Clavella adunca *8. *Copilia quadrata *9. *Chondracanthus zei *10. *Phyllothyreus cornutus *11. *Acanthocyclops vernalis *12. *Sapphirina ovatolanceolata *13. *Chondracanthus ornatus *14. *Corycaeus obtusus *15. *Euaugaptilus filigerus *16. *Monstrilla longispinosa *17. *Sphyrion lumpi *18. *Caligus elongatus *19. *Lernaeocera branchialis *20. *Oithona nana *21. *Sapphirina auronitens*. Sources: 1: [104]; 3, 15: [105]; 4, 5, 6, 8, 12, 14, 16, 20, 21: [106]; 7, 9, 10, 13, 17, 19: [107]; 11: [108] 2 & 18 original images, 2 drawn from photo taken by Jonathan Martin, Simon Fraser University.

Copepods are more abundant than any other group of multicellular animals, including the hyper-abundant insects and nematodes [3]. They pervade the majority of natural and man-made aquatic systems, inhabiting a domain that extends from the nutrient-rich black oozes of abyssal ocean depths to the nutrient-poor waters of the highest mountain tarns. Swarms of copepods can reach densities of up to 92,000 individuals L^-1^[4]. Some species have escaped traditional aquatic habitats, and live in rain forest canopies, leaf-litter, hot springs, between sand grains, in hyper-saline waters (~200 ppt) and in caves, as well as in symbiotic associations with other animal and plant species. Deeply divergent morphologies are found in relation to free-living or parasitic lifestyles, with some groups appearing classically "arthropodan", and others unrecognizable as such (Figure 1).

As the dominant secondary producers of the sea, copepods are the linchpin of aquatic food-webs. They consume microorganisms and are preyed upon by higher trophic levels, including fish and whales. In particular, they serve as primary prey for early life history stages of many fish species of economic importance [5], such as cod, herring, anchovy, flounder, and salmon. Copepods contribute significantly to many marine and freshwater ecosystem services, which have an estimated value of 22.6 trillion USD per annum [6]. For example, fish provide more than 2.9 billion people with more than 15% of their daily animal protein, and fisheries generate a net export value of $24.6 billion per annum for developing countries [FAO Newsroom (2006) http://www.fao.org/Newsroom/en/news/2006/1000301/index.html]. Copepods critically support this marine fish production, and therefore play an important role in the nutrition, health and well-being of people who have little access to other sources of animal protein. Through their vertical migrations between surface and deeper waters, copepods also play a major role in carbon transfer into the deep sea and thus to the global carbon budget (reviewed in [7]). Copepods are sensitive indicators of climate change, with warming ocean temperatures affecting copepod community structure, abundance, distribution and seasonal timing (e.g., [8]). In turn, changing copepod distributions have resulted in reduced recruitment and productivity of regional fisheries, such as the North Sea cod stocks (e.g. [5]).

Copepods harbor a wide range of human and fish pathogens. Pathogenic bacteria, such as *Salmonella *spp., *Enterococcus faecalis, Aeromonas *spp., and *Arcobacter *spp., as well as several pathogenic species of *Vibrio*, including *Vibrio cholerae *have been isolated from copepods [9-12], however, their role as vectors of waterborne bacterial pathogens of humans remains poorly understood.. Copepods are intermediate hosts for the Guinea worm, *Dracunculus mediensis*, which causes the debilitating disease dracunculiasis [13], as well as fish tapeworms (e.g. *Diphyllobothrium latum*) and anisakid nematodes that can also infect humans [14]. In addition to the status of copepods as carriers of pathogens, many parasitic and predatory copepods are in themselves pathogenic and have considerable impacts upon global freshwater and marine fisheries, with major economic consequences recognized primarily in aquaculture [15-17].

### Copepods: a resource for investigating fundamental biological processes

The extraordinary diversity of forms and life-strategies of copepods makes them very suitable for studies of a variety of fundamental biological processes that are of broad interest to the scientific community. As yet, however, little has been elucidated concerning the genomic architectures, transcriptional profiles or mechanisms controlling transcription that drive and underpin this diversity. Copepods could be used to examine questions of how genomic architecture differs among taxa and whether this limits or drives the observed morphological and ecological divergence, thereby influencing speciation events [18]. A related question is whether apparent change or simplification of form or function is reflected in the genome. For example, what is the driving force in the adaptation of copepods to a parasitic mode? Do copepod parasites necessarily possess "degenerate" genomes or are transitions in lifestyle accomplished by both gene losses and gains, or more simply by changing patterns of transcription?

Similarly, genomic data can provide answers as to what phenotypic and genomic characteristics have enabled major habitat transitions in free-living species, such as the move from the benthos to the pelagic environment, or from marine to freshwater biomes. There are a wide range of examples in both free-living and symbiotic copepods of closely related species that show distinct niche breadths in both their distribution and tolerance to environmental conditions. What then, can genomic and transcriptional data tell us about organismal and population responses to the environment in these cases? Since tolerance of environmental change, or lack of it, is ultimately genome-driven, one might ask whether genomic studies on the physiological responses to stress, could also provide tools for monitoring or predicting organismal or population responses to climate change in terms of genome structure.

The broad size range of eukaryotic genomes has been long recognized, but its causes and biological significance are still debated [19]. Some copepod species possess a large range in genome size within individuals as a result of excision of large amounts of DNA from the presomatic cell lineage during development [20]. The streamlined somatic genomes and dramatically augmented germline genomes observed in such species may provide a useful study system for understanding genome organization and mechanisms for altering genome size.

The monophyly of some orders is doubted, and the number of orders is defined to be between eight and eleven [21]. Thus, it is difficult to confidently choose orders whose phylogenetic position is near the root of the copepod lineage when constructing phylogenetic hypotheses across the major arthropodan lineages. Phylogenetic studies are increasingly employing large data sets of nuclear protein or transcript sequences [22,23], to resolve relationships among major arthropodan and ecdysozoan lineages, but to date have not elucidated the phylogenetic position of copepods [22]. Improved resolution of relationships in the future may require the use of phylogenomic approaches that compare large portions of genome or transcriptome sequences. For these reasons it seems necessary to make investments in the provision of large scale genomic resources for several taxa, and the choice of these taxa should be informed by their phylogenetic position relative to other copepods.

Genomic studies of copepods are relevant to many areas of fundamental and applied research. In particular, knowledge of the mechanisms underlying host-parasite relationships and features such as drug resistance can help to increase the sustainability of wild and cultured fisheries through development of improved chemotherapeutants, vaccines, and integrated pest management strategies. Historically, much of the fundamental research on plankton composition, population dynamics and response to environmental factors was driven largely by the need to characterize the impact of plankton on fisheries. Until recently such work was undertaken in the absence of molecular tools, however, genomic technologies are now providing new kinds of information as well as substantially decreasing the time interval between sample collection and analysis.

Aspects of symbiosis and parasitism, biological invasion, diapause, and genome size and reorganization deserve special mention in the context of copepod genomics. We discuss these topics below, and provide examples of how genomic tools might be harnessed to study these problems.

## Discussion

### Genomic insights into symbiosis and parasitism

One of the most extraordinary aspects of copepods, and one of the features that makes them so interesting for a range of genomic, functional genetic and transcriptomic studies, is their astounding capacity to form associations with other organisms. Nearly half of all known copepod species live in such associations [24]. Boxshall [pers. comm.] estimated that ~4152 species from 109 families are symbiotic or parasitic and suggested that there have been 11 or more independent origins for symbiosis/parasitism within and across the various orders, with a minimum of seven independent colonization events in fish. These associations range from so-called micro-predation, where species opportunistically snatch meals from their associates, to fully endoparasitic relationships in which the parasite is completely enclosed within the host and intimately associated with it. These features make copepods particularly suitable for studies of the changes in genome structure, such as gene loss and gain, that accompany the transition to a parasitic lifestyle, especially in situations where the free-living ancestral forms and newly parasitic forms retain similar morphologies and still live within a constrained habitat e.g. *Eucyclops bathanalicola *inhabiting Lake Tanganyika, the only parasitic member of an otherwise free-living copepod clade [25]. The availability of free-living and closely related parasitic forms for genomic study may allow answering of questions on the need for pre-adaptations to facilitate the transition to symbiotic modes of existence and also questions of the existence of key stepping stone hosts/associates in the multiple independent transitions to parasitism. Such questions are already being tackled in nematodes using genomic resources [26]. Development of novel functions, such as the ability to immuno-modulate or to stimulate hosts to directly support their survival, is also associated with the move to parasitism, as are the concomitant processes of speciation and adaptive radiation. Comparative genomic analysis and transcriptomic studies would allow a fuller exploration of the strategies by which copepod genomes evolve specializations to particular host-associations, as well as contribute to advancing our broader knowledge of parasite evolution. These types of studies have been conducted for other parasites, such as the causative agents of leishmaniasis (*Leishmania *spp.) and malaria (*Plasmodium *spp.) and have greatly contributed to understanding of their biology [27,28]. In another ecdysozoan, the filarial nematode *Brugia malayi*, genomic studies have indicated that up to 20% of predicted gene models are specific to the species and this has led to the suggestion that such genes may represent a pool of genes associated with defense/interaction with insect and human hosts [29].

Parasites have often been portrayed as degenerate versions of free-living forms, due to commonly observed features, such as morphological simplification. However, it has been suggested with respect to parasite genomes, that rather than being degenerate, they represent "...not the dustbins of history but the jewels of evolution" [30]. For example, while their ability to obtain a variety of resources from the host(s) may make some genomic features redundant, the relationship with the host is rarely one of passive nutrition, even for apparently morphologically simplified endoparasitic species such as *Sarcotaces *sp. (Figure 1 species 2). Kurland and colleagues [31] (pg 1013) suggest that "genome reduction and cellular simplification are hallmarks of parasites and symbionts", however, this may apply more to prokaryote and eukaryote intracellular parasites (e.g. see review by [32]) than to eukaryote ectoparasites and non-intracellular endoparasites. Copepod genome resources are thus far insufficiently developed to examine such questions, while genomic studies on ticks, which can similarly have longer term associations with hosts rather than taking quick meals, shows evidence for gene duplication and hence genome expansion. Like ticks, many copepod parasites are able to actively direct host physiology and have been shown, for example, to directly immuno-modulate fish hosts [33] or cause associates to build costly structures that favor the parasite [34]. Such abilities require genomic and transcriptional adaptations that will be understood only when genomic resources to conduct inter- and intra-species comparisons are available. In ticks, duplication of genes within a given gene family may perform a number of functions including increasing expression levels of anti-host products, allowing targeting of multiple related host defensins or the same host molecule in multiple hosts, and providing antigenic variation to avoid host attack while affecting the same host target [35]. Gene duplication may also provide for differential function between parasite stages or states [35].

Many symbiotic/parasitic copepod groups have switched host phylum in the course of their evolutionary history [36], and the question of how such switches occur remains an important one, as it also informs wider questions of adaptive radiation and the nature of speciation. Monstrilloids (Figure 1 species 16) for instance, whose ancestral adult forms are considered to have been ectoparasites of fishes, are now known to display a free-living adult and a fully endoparasitic invertebrate-associated larval stage. In addition to the radical host switch, the group has also undergone major changes in functional morphology and life-history strategy, such that Huys et al. [36] (page 376) consider this combination of adaptations to be "probably unique in metazoan parasites". Because of their many and taxonomically varied associations, copepods lend themselves to the study of horizontal gene transfer between microbial host-associates and the copepod, as occurs in plant, fungal and animal nematodes [37] and between hosts and copepod symbionts, a relatively little explored area. Complete copepod genome sequences would provide a key resource for such studies, enabling the inference of gene trees to employ phylogenetic incongruence as a criterion for detecting horizontal gene transfer (e.g. [38]).

One of the most interesting correlates with the transition to a wholly parasitic state is the change in body morphology. While some ectoparasitic adult stages closely resemble their free-living counterparts, many mesoparasitic/endoparasitic groups undergo an extravagant metamorphosis from the juvenile to the adult stage that renders them almost unrecognizable as arthropods (e.g. Figure 1 species 2). Genomic analysis could help us understand these changes by uncovering patterns of gene expression and regulation that occur at individual stages during development and metamorphosis and that result in these radically different adult morphologies.

Parasitic copepods also have major impacts on wild and cultured fisheries. As an example, caligid copepods (sea lice) are responsible for disease-related economic losses to marine salmoniculture that exceed $430 million worldwide per annum [17]. Sea lice have also been suggested to be directly or indirectly responsible for declines in wild salmonids. The control of parasitic copepods in aquaculture can involve the use of chemotherapeutants and as a result, some populations have developed resistance to treatment, a situation mirroring that observed in insect pests and perhaps unique among aquatic arthropods. Functional genetic studies are already providing insights into the basis for drug resistance in copepods (e.g. [39]) and into the mechanisms that copepods employ to avoid host immune responses (e.g. [33]). These observations increase the prospect of improved control of parasites in finfish culture systems. Gene knock-down studies in *Lepeophtheirus *using RNAi provide a powerful tool [40] to understand the function of individual genes, with attendant prospects for novel control strategies including development of vaccines or new chemotherapeutants. Functional genetic and transcriptomic studies also offer tools for monitoring the development of drug resistance in treated populations that are more sensitive and consistent than existing bioassays. Instead of measuring death or debilitation as outcomes of treatment, such tools allow measurement of direct or indirect response markers with a continuous distribution. New high throughput sequencing technologies which allow rapid simultaneous sequencing of millions of transcript or genome fragments per run [41] can similarly offer opportunities for detecting genomic markers for important traits such as drug resistance, which may then be used to develop parasite control strategies. Furthermore, genomics offers a powerful and innovative way to support the development of new therapeutants, as well as to identify novel compounds, such as immunomodulators, produced by parasitic copepods that are of scientific and/or medical importance. Identification of parasitic copepod orthologues of genes that are targets of therapeutants in other animals, especially those that are sufficiently different from that of their hosts, will help to identify and prioritize alternative therapeutants.

### Genetic mechanisms underlying biological invasions

Invasive species pose major threats to biodiversity, ecosystem integrity, agriculture, fisheries, and public health, with economic costs of nearly $120 billion per year in the US alone [42]. Understanding factors that allow some species to invade is crucial for mitigating and managing environmental impacts. Recent studies show that many invaders are crossing biogeographical boundaries into new habitats, and that evolutionary responses are often critical for these successful invasions [43-45]. Copepod invasions are a common and global phenomenon, the implications of which are poorly understood. Copepods generally comprise the most abundant and diverse taxonomic group within ship ballast water, and are thus transported worldwide in extremely large numbers [46]. Given the high number of pathogenic species found associated with copepods [9-14], copepod invasions could have important implications for dissemination and transmission of pathogens.

Invasive copepods provide particularly valuable models for exploring fundamental mechanisms of niche evolution. Frequent habitat shifts and short generation times make copepods amenable for analyzing the evolutionary and physiological mechanisms that underlie radical habitat transitions. As copepods are small and many species could be reared in the laboratory for several generations, they could be used for quantitative genetics and selection experiments, as well as association studies, to understand patterns of trait evolution and association between genes and traits. For example, within the past century the copepod *Eurytemora affinis *has invaded freshwater habitats from saline sources multiple times independently throughout the Northern Hemisphere [47]. Common-garden experiments have shown that these invasions have been accompanied by evolutionary changes in physiological tolerance, performance, and plasticity [48-50]. Most notably, freshwater populations have experienced evolutionary shifts in ion transport mechanisms, including increased activity and expression of the ion uptake enzyme V-type H^+ ^ATPase [50]. Modifying salinity alone during laboratory selection experiments recapitulated the evolutionary shifts in V-type H^+ ^ATPase activity observed in nature, providing strong support that salinity is a factor imposing selection in the wild [50]. Moreover, parallel evolutionary shifts were found in ion-motive enzyme activity and expression (V-type H^+ ^ATPase, Na^+^/K^+^-ATPase) across independent invasions [50]. In addition, a study using cDNA microarrays revealed parallel evolutionary shifts in expression of multiple genes and gene classes, including cuticle proteins, chaperones, cytoskeletal proteins, and ribosomal proteins, during independent invasions into freshwater habitats [50]. The parallel shifts suggest that shared genetic mechanisms might be implicated during these repeated evolutionary events.

As certain copepod species can be crossed in the laboratory, hybrid crosses between inbred lines, in conjunction with high-throughput sequencing, could be used to help determine whether evolutionary shifts in gene expression are the result of *cis- *or *trans-*regulatory changes in expression [51,52]. Such insights could provide invaluable information on the specific targets of selection and the causal mutations underlying evolutionary shifts in gene expression during independent invasion events. The latter would provide insights into the degree to which evolutionary pathways are labile or constrained during invasions. Moreover, as selection acts most strongly on genes underlying functionally important traits, identifying the genomic targets of natural selection during habitat invasions could reveal the traits that are critical for habitat shifts and address core questions regarding mechanisms of niche evolution. This general approach could also be profitably applied to understand other major habitat transitions within the Copepoda.

### Resurrection ecology and genetic regulation of diapause

Diapause is a life history trait common to many marine and freshwater free-living copepod species and is shared with many other arthropod groups. Duration of diapause varies from only a few months in juveniles of cyclopoid species to centuries in calanoid eggs. The biological significance of long-lived diapause eggs (> 300 yrs in some species; [53]), however, remains to be established. The deposition of diapause eggs in lacustrine and coastal marine sediments provides unique access to genetic archives of past populations representative of historic genotypes. The field of resurrection ecology, which has focused, to date, on the water flea *Daphnia *(e.g. [54]) has the potential to significantly advance our understanding of evolutionary responses to local conditions, through the study of genetic adaptations to environmental change. These eggs are an important resource from which we can directly observe fitness traits of animals adapted to past environmental conditions [55,56].

Although seasonal and environmental diapause patterns have been described for numerous copepod species [57], the suites of genes that are involved in diapause remain largely unexplored. In a number of arthropods, genes from the family of heat shock proteins are upregulated during diapause, acting as chaperone molecules against environmental stressors, e.g. temperature and anoxic conditions occurring during diapause [58-61]. In copepods, however, only one attempt has been made to employ genomic-related techniques to study diapause. Tarrant *et al*. [62] applied suppression subtractive hybridization gene libraries (SSH) and quantitative PCR (qPCR) to characterize gene expression in active and diapausing populations of *Calanus finmarchicus*, in order to describe the physiological regulation of dormancy. Using these techniques the authors were able to identify genes that were differentially expressed in these populations, including several that are involved in lipid synthesis leading up to dormancy and the chelation of metals during diapause. Identification of such genes is an important first step in the understanding of regulation and timing of diapause. The diapause trait itself is strongly dependent upon environmental cues such as temperature and/or photoperiod [57] and thus could be especially impacted by climate change. The significance of the genomic regulation and timing of diapause to a key group of organisms in the aquatic food web, is indisputable. This is particularly the case given the possible dramatic consequences of shifts in the timing of copepod diapause. Such shifts affect fish prey availability and recruitment (match-mismatch hypothesis [63]), and have critical downstream impacts on major coastal fisheries in marine systems.

### Genome size, reorganization, and co-adaptation

The dynamic nature of genomes is becoming increasingly evident in copepods and other eukaryotes and is challenging established views of how genomes evolve [64]. Large differences in genome content over the course of the organismal life cycle, reshuffled gene arrangements in the mitochondria, and the role of co-adaptation between nuclear and mitochondrial genomes during speciation events are areas where the molecular details of genome processes could be informed using genomic tools.

Embryonic chromatin diminution, the selective excision of large amounts of heterochromatic DNA from presomatic cell lineages, provides a dramatic example of augmentation of the germline genome and raises questions regarding the source of the increased amount of DNA and its relevance to the biology of the organism. For example, in *Mesocyclops edax *the somatic and germline nuclear DNA contents are ~ 3 and 30 Gb, respectively, while in *Cyclops kolensis *the DNA contents are ~1 and 75 pg, respectively [20,65]. The first studies to characterize the sequence identity of these streamlined somatic and "obese" germline genomes suggested preferential elimination of some microsatellite sequences and the DNA fragments located between microsatellites (e.g. [66,67]). Wyngaard and colleagues [20] posed two alternative mechanisms to account for the augmented germline genomes: (1) repetitive endocycles (repeated cycles of endonuclear DNA replication without intervening mitoses), and (2) proliferation of genetic elements in the germline genome. Data supporting the endocycle hypothesis are gleaned from thymidine labelling studies that reveal synthesis of DNA without intervening mitoses in *M. edax *germline nuclei containing ~ 9-30 Gb [65,68]. The alternative explanation hypothesizes the proliferation of genetic elements, which is plausible based on the population biology of genetic elements and their influence on genome architecture [69] and the contribution of transposable elements to variation in genome size among closely related taxa [70]. Chromatin diminution may serve to remove selfish genetic elements that have proliferated in the germline genome before their deleterious effects can impact the somatic genome. Such a scenario has been posed for ciliates that possess a similar form of chromatin elimination [71]. Ciliates, some parasitic nematodes, and Japanese hagfish all possess chromatin diminution, although certainly not of the same type and origin as described in copepods [72]. Of the taxa possessing chromatin diminution, the population biology and ecology are well known only for the copepods, enhancing the likelihood that any fitness consequences of chromatin diminution are more likely to be elucidated in this group. These genomic modifications are only one of a large array of models of genome size augmentation prevalent in other eukaryotes, can have far-reaching consequences on genome architecture and evolution, and warrant attention [64]

Another example of genome reorganization among copepods concerns the variable order of genes in the mitochondrial genome, a trait that is often conserved among vertebrate groups. Although complete mitochondrial DNA sequences are only available from five copepod genera, existing data show that copepod mitochondrial genomes have large-scale gene rearrangements relative to each other as well as to the "typical" arthropod mitochondrial genome [73-75]. For example, in *Tigriopus *all genes are encoded on the same DNA strand. In four other studied species, however, two for which the full mitochondrial genome sequence is known (*Lepeophtheirus salmonis and Calanus sinicus*) and two for which only partial mitochondrial sequences are available (*Eucalanus bungii *and *Neocalanus cristatus*), both DNA strands contain coding regions [74,76]. Since these rearrangements result in the need for multiple promoter sites, studies of mtDNA gene expression in these different groups may provide new insights into how gene arrangements impact mitochondrial function.

Copepods have also served as model systems for understanding genomic co-adaptation. When gene flow among populations is restricted, natural selection can result in adaptation to local environments. Selection also acts within the genome to retain sets of alleles that interact well and produce the fittest individuals (regardless of external environment), a process known as genomic co-adaptation [77]. When divergent populations are hybridized, breakdown of co-adaptation is manifest in poor hybrid fitness, often viewed as a first step in the formation of new species. The tidepool copepod *Tigriopus californicus *has become a model system for studies of co-adaptation and hybrid breakdown. Ellison and Burton [78] found that hybridization leads to altered transcription and replication of *T. californicus *mtDNA, a consequence of disrupted co-adaptation between nuclear and mitochondrial genomes. Edmands *et al*. [79] found evidence for maladaptive combinations of alleles within natural *T. californicus *populations, presumably due to fixation of deleterious mutations by genetic drift in small populations. Most recently, protein-coding regions (the transcriptome) of two divergent populations of *T. californicus*, obtained using 454 pryosequencing, revealed evidence of positive selection that potentially plays a role during the early stages of reproductive isolation [18]. Genome-level studies of hybrid individuals promise to elucidate the genetic interactions that underlie hybrid breakdown and may provide clues to the process of speciation. Similar conclusions about the role of selection in the formation of hybrids were gleaned from the first nearly complete map of a copepod genome derived from hybrids between two *T. californicus *populations [80].

### Using genomic tools to detect responses to the environment

Understanding responses of copepods to environmental conditions is one important research focus that has been limited by available techniques. To date, relatively few research groups have used genomics-related technologies to study zooplankton responses to their environment. However, recent insights from the genome of the freshwater cladoceran *Daphnia pulex *suggest that gene duplication and differential expression can be an important avenue enabling flexible phenotypic responses to ecological challenges [81]. Despite possessing a diminutive genome of about 200 MB, the estimated number of genes of this zooplankter is over 30,000, about 10,000 more than that found in humans. More than a third of these genes have no known homolog. The elevated gene count in *D. pulex *has been attributed to gene duplication and retention, which have allowed distinct expression patterns to evolve across paralogs. Known for its morphological and physiological plasticity in responding to environmental insults (e.g. heavy metals) and ecological conditions (e.g. predation), *Daphnia *has been used as a model for both toxicological and ecological studies (e.g., for the Environmental Protection Agency; [82]). The eco-responsive genome of *Daphnia *provides a vision of the nature and extent of the insights that might be obtained from comparable studies of copepod genomes. Because copepods have greater taxonomic, life history, and habitat diversity, it may be possible to obtain insights into how genome architecture and expression patterns facilitate biological response across a wider range of environmental conditions. In addition, copepods lack parthenogenesis and thus may provide a more appropriate model for other obligate sexually reproducing organisms.

Within copepods, the majority of studies conducted using genetic and genomic technologies to study responses to environmental conditions have been in toxicology. The ability to detect copepod responses to environmental pollutants has been limited by a lack of specific assays for most traits of interest, and has necessarily focused on responses at the individual or population level. The use of genomics-related technologies has several advantages over traditional methods, which often rely on whole-animal assays and endpoint measurements such as time to death, and allows for the identification of molecular pathways involved in any given response and enables monitoring of sub-lethal effects at the genetic level. Recent examples of work in this area include candidate gene studies on the effects of trace metals in *Tigriopus japonicus *[83], and the effect of oil exposure on gene expression in *Calanus finmarchicus *[84]. Hansen et al.'s [84] work on *C. finmarchicus *is particularly timely in light of the large volumes of crude oil spilled in the Gulf of Mexico in 2010. This study demonstrated that levels of glutathione S-transferase (GST) and cytochrome P-450 330A1 (CYP330A1) changed significantly in response to the presence of dispersed petrogenic oil in seawater, with differential responses being observed in copepod lipid levels/reproductive state. Recently there has been a transition in copepod studies, from the use of single genes to investigate traits of interest to the use of large-scale transcriptional profiling. Large-scale transcriptional profiling provides the capability to simultaneously assess the involvement of thousands of genes in a particular biological process, allowing new pathways, mechanisms of control, and relationships between different genes to be discovered. Examples of such an approach are the use of microarrays in trace metal risk assessment using *T. japonicus *[85] and the use of subtractive hybridization (SSH) to investigate effects of oil pollution on *C. finmarchicus *[86].

In marine ecology, genomics-related technologies could facilitate research to detect copepod responses to environmental change at the levels of genes, whole organisms and populations. One important contribution of genomics could be the development of molecular markers for physiological stressors, such as starvation, infection (parasitic, bacterial, viral), senescence, and thermal stress (climatic change), which could provide insight into how these processes influence growth and mortality in natural populations. Studies that document the frequency, extent, and sensitivities of copepods to stress from a variety of sources would provide important insights into the proximate causes of seasonal changes in population abundance and the factors that limit biogeographic range, and could also prove useful in predicting population responses to climate change (*e.g*., population forcing through thermal stress). Recently, several laboratories have started to develop techniques to investigate copepod gene expression in order to better understand stress in response to environmental conditions. Voznesensky et al. [87] used a qPCR approach to quantify expression levels of hsp70 in response to thermal stress in the abundant North Atlantic copepod, *C. finmarchicus*, and found that increases in gene expression due to migration or transport across natural thermal gradients should be detectable in assays of field populations. More recently, Tarrant et al. [62], among others, have used gene expression studies to begin identifying genes that are regulated seasonally. Finally, Christie et al. [88] have generated approximately 10,000 expressed sequence tags (ESTs: Table 1) and developed a 1000-probe microarray for *Calanus finmarchicus *that is being used to study gene expression patterns associated with season and location under natural as well as experimental conditions.

**Table 1:** Publicly available sequence data for copepods

	Core nucleotide	Expressed sequence tags	Mitochondrial genome sequence	Nuclear genomic sequence (chromosome/whole genome)	Scientific relevance of species
Hexapoda (insects)	3,076,212	4,496,444	235	51/20	

Copepoda (Total)	15,316	207,282	8	0/0	

*Lepeophtheirus salmonis*	4,345	129,250	2	0/0	Parasite of wild and farmed marine fish, economically and ecologically important

*Caligus rogercresseyi*	1,610	32,037	0	0/0	Parasite of wild and farmed marine fish, economically important

*Caligus clemensi*	1,227	14,806	0	0/0	Parasite of wild and farmed marine fish

*Lernaeocera branchialis*	1	14,927	0	0/0	Parasite of wild marine fish

*Calanus finmarchicus*	48	11,461	0	0/0	Key marine zooplankton species

*Tigriopus californicus*	920	4,801	3	0/0	Tidepool copepod used as a model system in evolutionary genetics and ecotoxicological research

Publicly available sequence data for copepods and for insects, the other hyper-abundant arthropod taxon, which have received far more attention in genomics studies (source: Taxonomy Browser, GenBank release 184.0, accessed July 22nd, 2011). Data include only those copepod species for which there are greater than 1000 core nucleotide or EST sequences. While data resulting from shotgun genome sequencing exist for *L. salmonis *(475,815 sequences) no complete genome assembly has thus far been conducted.

Genomics-related technologies could also be used to assess shifts in copepod community composition or biogeographic range in response to environmental conditions. Determination of the composition of bulk zooplankton samples using traditional methods is a time consuming process that requires broad expertise in taxonomy to ensure correct species identification. Community metagenetics and DNA barcoding provide a high throughput alternative to visual assessment of the diversity of plankton assemblages, with more conventional genetic approaches typically used to identify eggs and larval stages, to detect cryptic species, and to study species distributions (e.g. [89-91]). Although metagenomic approaches (see [92] for a lucid commentary) are now being used to assess diversity of aquatic microbial communities (e.g. [93]), their application to copepods and other metazoans may be limited due to the larger genome size and complexity of eukaryotes. However, the metagenetic approach of high throughput sequencing of biodiversity tags (e.g., hypervariable regions of ribosomal genes) to assess diversity of protistan assemblages (e.g., [94]) is promising, and a similar approach could be developed for metazoan zooplankton based on the DNA barcoding gene mtCOI (mitochondrial cytochrome oxidase I). Use of high-throughput sequencing technologies could revolutionize plankton identification and the assessment of ecosystem health by allowing comprehensive and rapid surveys of plankton community composition.

### Resources and strategies

#### Existing copepod sequence resources

Currently no assembled genomes exist for the Copepoda, although some limited shotgun sequencing of the genome of the salmon parasite *Lepeophtheirus salmonis *has been undertaken recently. Thus, with the exception of 8 mitochondrial genome sequences, the publicly available genomics resources for copepods consist primarily of expressed sequence tags (EST's) that have been obtained from normalized, non-normalized or subtracted libraries. Of these, the largest numbers have been obtained for the parasitic species *L. salmonis *and *Caligus rogercresseyi*. These species have received attention due to their economic importance to salmon farming. Although many of these data were initially generated to support specific projects related to their control and treatment, they have enabled a wide range of studies on ecology [95], development [96], drug resistance [39] host-pathogen interactions [33] and the characterization of gene families of interest [97]. The second largest number of sequences belongs to the key free-living planktonic calanoid species *C. finmarchicus*. These genomic resources have facilitated the development of cDNA and oligo-based microarrays for *L. salmonis *and *C. finmarchicus *that are being utilized to investigate a variety of aspects of their biology, such as their host-interactions [96], and responses of planktonic species to environmental changes associated with season or depth [88]. These resources have also provided the building blocks for techniques that are fundamental to deeper understanding of gene function, such as individual gene knock-out through RNA interference [40].

When compared to other arthropod groups such as the insects, available genomics resources for copepods are extremely limited, especially given the dominance and importance of this group in aquatic ecosystems (Table 1). Currently, the most extensive arthropod genomic resources are those developed for the fruit fly *Drosophila melanogaster *and other *Drosophila *spp. Genome sequencing and assembly and consequent downstream genomic studies have revolutionized genomic studies in this species and have, moreover, proven highly informative with respect to other metazoans including humans. A précis of the enormous advantages that genome resources have provided for the *Drosophila *research community is provided by Ashburner *et al*. [98] and current work seeks, among other tasks, to expand on this knowledge by providing a comprehensive identification of the sequence-based functional elements within the *D. melanogaster *genome as part of the model organism Encyclopedia of DNA Elements (modENCODE) project [99]. Recent sequencing of other arthropods, including mosquito disease vectors (e.g. [100]) and other insects with high economic/environmental importance, e.g., honey bees [101], have also advanced genomic and biological studies in these species. Genomes of aquatic arthropods are limited to those of *Daphnia *spp. [81], which like copepods, bring to the table an extensive history of biological, behavioral and ecological research that considerably widens the benefits, in terms of relating genome structure to environmental forces and biological function, that may be obtained from provision of genomics resources.

#### Why adopt copepods as genomic models?

It is clear from the above discussions that copepods can be used to address many fundamental and applied biological problems. While this might be true of many groups, the ubiquity, importance, and diversity of copepods, allied with their suitability for laboratory studies, makes them potentially highly informative as model organisms across a wide range of research disciplines. Their utility, however, is dependent upon the generation and availability of suitable genomics resources.

**Figure 2:**
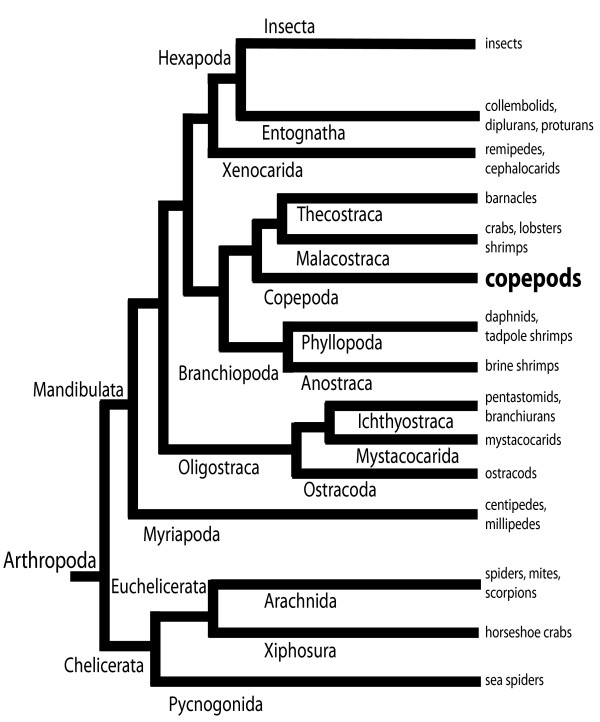
**Simplified phylogram showing the position of the copepods with respect to the other major arthropod taxa**. This topology is based on the largest data set available, 41 Kb of single-copy nuclear protein coding genes, and is modified after [1].

The two dominant invertebrate genetic model organisms, the nematode *C. elegans *and fruit fly *Drosophila*, are members of other hyper-abundant and hyper-diverse clades, leaving the copepods as the only hyper-diverse ecdysozoan group, for which a full or draft genome assembly does not exist. Furthermore, the only crustacean genomes sequenced and assembled to date are those of the branchiopods *Daphnia pulex *and *D. magna *so that the diverse crustacean clade itself is highly under-represented in terms of genome models (Figure 2). *Daphnia *was listed as the 13th official model organism for biomedical research by the National Institutes of Health, and the diverse Copepoda harbor many species that will similarly prove highly informative to biomedical research.

#### Criteria for candidates for large-scale resources

Selection of candidate species for whole-genome sequencing is complicated by the considerable genetic diversity among orders, as well as within single species or species complexes (e.g., [102]). Such high divergence emphasizes the need to target several copepod species in parallel for the development of genomics resources. Still, for many taxa, the key criteria typically used for choosing such candidate species [58] are lacking. The haploid genome size of copepods http://www.genomesize.com covers a broad range, from 0.14 pg -12.46 pg (compared to a human = 3.5 pg) and clearly, with other factors being equal, the smaller the genome, the more rapid and inexpensive the sequencing and assembly. Choosing species that possess a conventional genetic system will also increase the likelihood that sequencing and assembly of the genome is straight-forward. Widely distributed species ease access to local populations for researchers who collectively bring a broad array of interests and expertise to the field. Many species are already routinely cultured (e.g. *Temora longicornis, Eurytemora affinis, Centropages hamatus, Tigriopus californicus, T. japonicus, Tisbe biminiensis, Acartia tonsa, Mesocyclops longisetus, Lepeophtheirus salmonis*) although the parasitic species still require live hosts for culture. A number of inbred lines of copepods already exist (e.g. *E. affinis, L. salmonis *and *T. californicus*), which should be helpful in assisting genome assembly, promoting experimental consistency and helping dissection of particular traits such as chemotherapeutant resistance or salinity tolerance. Although molecular and computational techniques have allowed considerable progress to be made in the resolution of phylogenetic relationships among major ecdysozoan lineages (e.g. [22]), the complete absence of higher-order molecular phylogenies for the Copepoda impedes the choice of candidate species based on phylogenetic position. Perhaps, therefore, the choice of candidate species is best determined by the relative merits of the particular biological problems these candidates would address.

Genomic resources exist for species in the Harpacticoida, Siphonostomatoida, and Calanoida (see Table 1). Affordable and efficient comparative genomics, utilizing several copepod taxa and a combination of whole genome and EST analysis, is now feasible due to recent advances in sequencing technology. The concomitant decline in sequencing costs will likely alter the relative amounts of genomic data available to design efficient and successful sequencing projects for a particular species. Thus, any priority list of candidate species for large scale genomics studies is a moving target. If the goal is to understand the diversity of form and function in copepods, the evolutionary mechanisms responsible for them, and how this diversity is related to other major arthropod groups, it is clear that forms representing the diverse orders will require study. Similarly, if the goal is to understand symbiosis and parasitism, pairs of sister taxa that include free-living and parasitic forms will have to be compared. Alternatively, if the goal is to the reveal the genetic mechanisms involved in population differentiation and speciation, a single species such as *T. californicus *or *E. affinis *may suffice. It is more likely that suites of copepod species will be required for robust tests of most problems of general interest, whether the questions are fundamental or applied or whether they are ecological or evolutionary. The principal goal in the ongoing development of genomic resources for copepods should be the targeting of a diversity of species such that genomic resources facilitate research on diverse problems of global importance.

#### Sequencing Goals

In considering a whole genome sequencing project the advantages of a high coverage sequencing effort with subsequent assembly over low coverage/transcriptome sequencing effort need to be assessed. Assembly of the millions of short sequences provided by low cost sequencing is facilitated by availability of a pre-existing reference genome. In copepods, we do not yet know which species might be representative (ancestral), nor do we know the extent of differences in genomic structure, gene order, repeats and other features of the genome, which might be relevant to sequence assembly. The current pace of technological change precludes a discussion of sequencing strategy in this paper. However, a number of questions regarding sequencing of copepods might still be constructively addressed.

A genome does not need to be sequenced in depth and assembled to be useful (even the human genome remains incomplete). The level of coverage and contiguity required depends in part upon whether the objective is to examine a single species, a number of closely related species or an array of diverse forms. Furthermore, whether the purpose of the study is structural or functional genomics will have a bearing on the sequencing approach. Although whole genome sequencing has been the most rigorous method of choice for identifying SNPs (single nucleotide polymorphisms) when dissecting the origin and population structuring of traits of interest, the low cost RAD-Seq method (restriction-site-associated DNA sequencing) may prove to be just as informative [103]. Whole genome sequencing may still be the method of choice, however, for identifying other structures such as repeats and indels.

The power of a highly assembled genome is that it can potentially provide a framework for the rapid assembly of genomes from the same or closely related species and more importantly, a reference for subsequent studies involving high throughput sequencing that provide high numbers of short reads. It is extremely likely that the divergence among copepod lineages is so great that a full genome sequence of one copepod species will not suffice as a scaffold for assembly of others, save for quite closely related species.

## Summary

Copepods comprise an extremely abundant, diverse and ecologically significant group, for which few studies have fully exploited the power of genomic technologies. Provision of multiple genomes or large-scale resources for a number of species could provide unparalleled insights into biodiversity and evolution. This single taxon has evolved into a diverse group with multiple convergent instances of the evolution of parasitic associations, a transition from benthic to pelagic life-styles, and invasions into extreme habitats (deep ocean, caverns, polar regions). Even the sequencing of a single copepod genome would provide a basis for evolutionary comparisons, such as resolving relationships among major pan-crustacean taxa. Availability of large scale genomic resources also opens the door to a wide range of other "omics" studies. In addition, such resources will: 1) allow research groups that have limited resources to begin applying molecular methods in their research, 2) greatly extend the number of traits we can study to better understand copepod biology and interactions with their environment, 3) enable researchers to develop tools to address many questions where more traditional methods have limitations, 4) enable development of standardized molecular techniques, providing a measure of uniformity and consistency to the measurement of biological properties of copepods, and 5) attract new researchers from diverse disciplines into the field. Development of copepod genomics resources must be supported by the education of present and future generations of scientists in genomics and bioinformatics, in order to ensure successful exploitation of these tools and resources. Current strategies must anticipate step-changes in approach, as well as providing resources to carry out further types of 'omics' studies on copepods.

## Competing interests

The authors declare that they have no competing interests.

## Authors' contributions

All authors contributed ideas during the Copepod Genomics Workshop held during the World Association of Copepodologists conference in Thailand 2008. JEB, DF, SCJ and GAW drafted the manuscript. JEB prepared the table and figures. All authors intensively revised subsequent drafts of the manuscript and read and approved its final version.

## Authors' information

James Bron is a marine biologist working in the area of cultured fish health and welfare and has particular expertise in host-parasite interactions between salmonid fish and their copepod parasites. He also has long-standing experience in the application of genomic methods, particularly transcriptomic analysis. He is currently a senior lecturer in the Parasitology Research Laboratory, Institute of Aquaculture, University of Stirling, Scotland.

Dagmar Frisch is an aquatic ecologist with special interest in the ecological genetics and the evolutionary ecology of crustacean zooplankton. She is a postdoctoral researcher at the University of Oklahoma Biological Station, USA.

Erica Goetze's areas of expertise cover the evolution and ecology of marine calanoid copepods, with her current research focused on understanding dispersal and gene flow in marine zooplankton populations. She is an assistant professor at the University of Hawaii at Manoa, Hawaii.

Stewart Johnson's research areas include aquaculture, aquatic animal health and biotechnology/genomics with special interest in the development of genomics resources for key aquaculture species. He is Head of Aquatic Animal Health at Fisheries and Oceans Canada, Pacific Biological Station, Nanaimo, British Columbia.

Carol Lee is an evolutionary geneticist focusing on the quantitative genetics, population genetics, and genomics of invasive populations, including copepods. She is a professor in the Center of Rapid Evolution (CORE) and the Department of Zoology at University of Wisconsin, Madison.

Grace Wyngaard's research focuses on copepod evolution, genome size, chromatin diminution and the phylogenetic relationship of copepods based on molecular and morphological characters. She is a professor in the Department of Biology at James Madison University, Virginia.
